# Electrophysiological alterations in a murine model of chronic coxsackievirus B3 myocarditis

**DOI:** 10.1371/journal.pone.0180029

**Published:** 2017-06-23

**Authors:** Sven Kaese, Robert Larbig, Matthias Rohrbeck, Gerrit Frommeyer, Dirk Dechering, Jan Olligs, Sabine Schönhofer-Merl, Rainer Wessely, Karin Klingel, Guiscard Seebohm, Lars Eckardt

**Affiliations:** 1 Division of Electrophysiology, Department of Cardiovascular Medicine, University of Münster, Münster, Germany; 2 The IfGH-Myocellular Electrophysiology, Department of Cardiovascular Medicine, University of Münster, Münster, Germany; 3 Interdisciplinary Centre for Clinical Research (IZKF), Faculty of Medicine, University of Münster, Münster, Germany; 4 Deutsches Herzzentrum and Medizinische Klinik, Klinikum rechts der Isar, University of Technology, Munich, Germany; 5 Zentrum für Herz- und Gefäßmedizin, Im Mediapark 2, Köln, Germany; 6 Department of Molecular Pathology, University of Tübingen, Tübingen, Germany; University of Minnesota, UNITED STATES

## Abstract

**Introduction:**

Coxsackievirus B3 (CVB3) is known to induce acute and chronic myocarditis. Most infections are clinically unapparent but some patients suffer from ventricular arrhythmias (VA) and sudden cardiac death (SCD). Studies showed that acute CVB3 infection may cause impaired function of cardiac ion channels, creating a proarrhythmic substrate. However, it is unknown whether low level CVB3+ expression in myocytes may cause altered cardiac electrophysiology leading to VA.

**Methods:**

Cellular electrophysiology was used to analyze cellular action potentials (APs) and occurrence of afterdepolarizations from isolated cardiomyocytes of wildtype (WT) and transgenic CVB3ΔVP0 (CVB3+) mice. Further, we studied surface ECGs, monophasic APs, ventricular effective refractory period (VERP) and inducibility of VAs in Langendorff-perfused whole hearts. All used cardiomyocytes and whole hearts originated from male mice.

**Results:**

Cellular action potential duration (APD) in WT and CVB3+ myocytes was unchanged. No difference in mean occurrence or amplitude of afterdepolarizations in WT and CVB3+ myocytes was found. Interestingly, resting membrane potential in CVB3+ myocytes was significantly hyperpolarized (WT: -90.0±2.2 mV, n = 7; CVB3+: -114.1±3.0 mV, n = 14; p<0.005). Consistently, in Langendorff-perfused hearts, APDs were also not different between WT and CVB3+ whole hearts. Within both groups, we found a heart rate dependent shortening of ADP_90_ with increasing heart rate in Langendorff-perfused hearts. VERP was significantly prolonged in CVB3+ hearts compared to WT (WT: 36.0±2.7 ms, n = 5; CVB3+: 47.0±2.0 ms, n = 7; p = 0.018). Resting heart rate (HR) in Langendorff-perfused hearts was not significantly different between both genotypes. Electrical pacing protocols induced no VA in WT and CVB3+ hearts.

**Conclusion:**

In CVB3+ mice, prolonged ventricular refractoriness and hyperpolarized resting membrane potentials in presence of unchanged APD were observed, suggesting that low level CVB3 expression does not promote VA by altered cardiac electrophysiology in this type of chronic myocarditis. These findings may suggest that other mechanisms such as chronic myocardial inflammation or fibrosis may account for arrhythmias observed in patients with chronic enteroviral myocarditis.

## Introduction

Coxsackievirus B3 (CVB3) is a non-enveloped virus with a single-stranded and positive polarity RNA genome and belongs to the picornavirus family of the enterovirus genus [1, 2]. Six serotypes of coxsackievirus of group B have been identified (CVB1-6) which cause diverse diseases as pancreatitis [3–5], meningitis or encephalitis [6] and myocarditis [1, 2, 5, 7–11]. Enteroviral infections due to CVB1-5 are present in around 5 million U.S. citizens and approximately 600,000 suffer from myocardial infection and myocarditis, mainly due to CVB serotypes 1,3 and 5 [1]. A wide range of patients with CVB3 infection (90%) and acute viral myocarditis are clinically unapparent or suffer from mild symptoms and may experience complete convalescence, whereas a certain proportion of patients present with rapid progress and severe cardiac dysfunction, ventricular arrhythmias (VA) or sudden cardiac death (SCD) [12]. Further, acute CVB3 myocarditis can merge into chronic viral myocarditis which finally results in dilated cardiomyopathy (DCM), as CVB3 antibodies can be detected in around 50% of patients with DCM [1]. In 20–25% of patients suffering from DCM, persistence of CVB3 genomes have been identified by PCR and *in situ* hybridization in cardiomyocytes [1, 7, 13–15]. These findings led to the hypothesis, that persistent virus infections may be involved in the pathogenesis of chronic myocarditis that finally results in development of DCM. Enterovirus-induced chronic myocarditis or DCM may develop as a consequence of acute myocarditis with subsequent insufficient regenerative mechanisms [16]. Acute enteroviral myocarditis is characterized by a high rate of viral RNA and protein expression and infiltration of the myocardium with inflammatory immune cells [7, 17]. Chronic viral myocarditis/DCM is associated with the persistence of CVB3 genome with low levels of virus replication, and a comparable positive-sense to negative-sense viral RNA ratio and persistent inflammation [1, 16]. Infectious CVB3 virus progeny cannot be detected in myocardial biopsy of patients with chronic viral myocarditis or DCM [1, 7].

However, there is an ongoing controversial discussion whether persistence of enteroviral genomes in myocardial cells contributes to chronic myocarditis resulting in development of DCM, or whether they simply represent remnants of a previous infection without significant pathological influence on chronic heart disease [18].

Previous studies have shown that persistent expression of a mutated copy of the full-length CVB3 genome, without formation of lytic infectious virus particles in transgenic CVB3deltaVP0 mice, exhibit a phenotype of dilated cardiomyopathy [18]. Hearts of these mice showed typical histopathological and echocardiographic characteristics of DCM like myocardial interstitial fibrosis, dilatation of the left ventricle, reduced systolic function, decreased left ventricular fractional shortening and increased end-diastolic and end-systolic dimensions of the left ventricle [18]. These alterations may contribute to arrhythmogenesis in this mouse model, as impaired left ventricular function is known as a predictor of VA and SCD [19]. Further, transgenic CVB3 mice showed disturbed excitation–contraction coupling, and reduced magnitude of isolated cell shortening [18]. In this study, Wessely et al. detected decreased [Ca^2+^]_i_ transients in isolated cardiomyocytes from CVB3 mice as compared to wildtype myocytes, while I_Ca_ current density was not affected [18]. In addition, a previous study showed that acute CVB3 infection of *Xenopus laevis* oocytes may cause impaired function of the cardiac ion channels I_Ks_ (α-subunit: KCNQ1, K_v_7.1), I_Kr_ (α-subunit: hERG1, hK_v_11.1) and I_CaL_ (α-subunit: Ca_v_1.2) which may create a potentially proarrhythmic substrate [2]. Another study further demonstrated that low level expression of CVB3 in cultured cardiomyocytes can induce cytopathic effects [17].

Although these studies found first hints that electrophysiological alterations may occur in acute and persistent CVB3 infection it is further unknown whether persistent low level CVB3 expression actually causes proaarhythmic alterations of cardiac electrophysiology leading to VAs. Another hypothesis is that other mechanisms like necrosis of myocytes, chronic inflammation and generation of myocardial scars may be relevant mechanisms of arrhythmogenesis in chronic myocarditis.

In order to study cardiac electrophysiological properties and vulnerability to ventricular arrhythmias in chronic myocarditis we used isolated cardiomyocytes and whole-heart preparations from transgenic mice expressing a replication-restricted full-length CVB3 cDNA mutant (CVB3ΔVP0) driven by the αMyHC promotor (CVB3+ mice). In the current study we show that in contrast to acute CVB3 infection [2], chronic low level CVB3 expression in chronic myocarditis seems not to alter cardiac electrophysiology in a proarrhythmic manner as we could not detect VA. These findings may contribute to our understanding of arrhythmogenesis in chronic CVB3 myocarditis and may suggest that low level CVB3 expression does not promote VA by altered cardiac electrophysiology and may support the hypothesis that other mechanisms like chronic myocardial inflammation over a longer period of time with necrosis and subsequent myocardial scars may predominantly contribute to VAs in chronic myocarditis.

## Methods

All experimental protocols were approved by the local animal care committee and conformed to the Guide for the Care and Use of Laboratory Animals published by the US National Institutes of Health (NIH Publication No. 852–3, revised 1996). The study was approved by the “Landesamt für Natur, Umwelt und Verbraucherschutz Nordrhein-Westfalen“. Reference number is 84–02.05.20.12.317.

### Animals

Generation of the CVB3 genome and characterization of the CVB3+ mouse model has been reported detailed previously [20, 21]. In summary, we used transgenic mice expressing a full length CVB3 cDNA mutant (CVB3ΔVP0) under the control of the α-myosin heavy chain (α-MHC) promotor [20, 21]. The CVB3 genome was mutated at the VP2/VP4 autocatalytical cleavage site in order to prevent formation of infectious viral progeny [18, 20, 21]. This mouse model mimics persistence of the CVB3 genome with CVB3 genome replication and protein expression [20, 21]. For confirmation of CVB3+ expression in CVB3+ transgenic mice and for identification of homozygous CVB3+ mice, we performed nested-PCR [20, 21]. The age of mice used for Langendorff and patch clamp experiments was 24.4 ± 0.4 weeks for wild-type (WT) and 24.0 ± 0.9 weeks for CVB3+. In our study, all used cardiomyocytes and whole hearts originated from male C57BL/6 mice.

### Cellular electrophysiology

We used collagenase/protease digestion to isolate ventricular cardiomyocytes from WT and CVB3+ mice as reported previously [22, 23]. Following isolation, we stored the dissociated cells for up to 6 h at room temperature in modified Tyrode solution, containing (in mM): 136 NaCl, 5.4 KCl, 10 Hepes, 1 MgCl_2_, 0.33 NaH_2_PO_4_, 1 CaCl_2_, 10 D-Glucose (pH 7.4 with NaOH). This solution was also used as the standard bath solution for electrophysiological recordings. We anaesthetized mice using *Ketamine* and *Xylazine* and removed hearts via thoracotomy.

#### Recordings of action potentials from isolated cardiac myocytes

To record cellular action potentials we placed the cells in an experimental chamber (0.5 ml) mounted on the stage of an inverted microscope (Zeiss Axiovert 200, Carl Zeiss, Oberkochen, Germany). A room temperature bath solution (20–22°C) continuously perfused the chamber. Patch electrodes were pulled from borosilicate glass (Harvard Apparatus, Holliston, MA, USA) on a Sutter P-97 horizontal puller (Sutter Instruments, Novato, CA, USA). The fire-polished electrodes had a tip diameter of 2–3 μm and a resistance of ~2MΩ when filled with the patch electrode solution, which contained (in mM): 127 KCl, 10 Hepes, 10 NaCl, 0.1 cAMP, 5 MgATP. This solution was used for experiments for recording action potentials. We used a HEKA EPC-10 patch-clamp amplifier (HEKA, Bellmore, USA) which was controlled by PatchMaster v2x53 software (HEKA, Bellmore, USA).

#### Pulse protocol

To record cellular action potentials, cardiomyocyte membranes were sealed with the borosilicate pipettes. After breaking, the current-clamp configuration was established and action potentials were recorded at 1,2 and 5 Hz. 16 action potentials were recorded at each frequency. The last four action potentials were analyzed for APD_90_ and membrane potential and all 16 action potentials were analyzed for the occurrence of EADs and DADs.

### Whole heart Langendorff

#### Preparation

WT mice and CVB3+ mice were anesthetized by using *Ketamine* and *Xylazine*. Mice were fixed to an operating table and thoracotomy was performed to remove the heart-lung package which was transferred into cooled, oxygenated and heparinized modified Krebs-Henseleit buffer. Attached tissue and lungs were excised and the aorta was cannulated. Afterwards, the cannula was connected to the Langendorff system (Hugo-Sachs, March-Hugstetten, Germany) and the heart was retrogradely perfused with 37°C warm modified Krebs-Henseleit buffer. The modified Krebs-Henseleit buffer contained (mmol/l): NaCl 116, CaCl_2_ 2.5, NaHCO_3_ 24.9, KCl 4.5, KH_2_PO_4_ 1.2, Glucose 9.9, MgSO_4_ 0.5 and EDTA 0.07. The buffer was equilibrated with a 95% O_2_ and 5% CO_2_ gas mixture. The cannulated hearts were attached to a vertical Langendorff apparatus and placed in a heated chamber to maintain 37° temperature of the hearts (Hugo-Sachs, March-Hugstetten, Germany).

#### Electrophysiological setup

An ECG was recorded from the epicardial surface. ECG signals were amplified and filtered with a bandwidth of 0.1 to 300 Hz (Hugo-Sachs, March-Hugstetten, Germany). Further, an electrode for recording of monophasic action potentials (MAP) was placed on the left ventricular epicardial surface. Custom made, miniaturized MAP catheters were fixed on electrode holders to ensure perpendicular constant contact and pressure to the epicardial surface in order to enable sufficient recording conditions. MAP signals were amplified and filtered (low pass 0.1 Hz, high pass 300 Hz). A pacing electrode was placed on the ventricular epicardial surface to apply electrical pacing protocols (Programmable Stimulator, Hugo-Sachs, March-Hugstetten, Germany). All signals were digitized and stored using the “IsoHeart” Software on a removable hard disc (Hugo-Sachs, March-Hugstetten, Germany).

#### Experimental protocol

After connection of the cannulated hearts to the Langendorff-setup, an equilibration period of 10 minutes was used for stabilization of the preparation and placement of the ECG and MAP electrodes on the epicardial surface. Subsequent, baseline heart rate was recorded. An electrical pacing protocol was applied to determine ventricular APD during fix-frequent stimulation, ventricular ERP and induction of ventricular arrhythmias. Hearts were paced with double diastolic threshold. Ventricular pacing with cycle length of S1 = 100, 120 und 150 ms was performed for 1 minute to enable steady state of ventricular APD. Studying ventricular ERP was performed by using a premature extra-stimulus (S2). After a train of 8 x S1 = 120 ms beats an S2 extra-stimulus was coupled. Each S2 cycle length (CL) length was applied two times to ensure reproducibility and was reduced by 2 ms during each pacing train. ERP was defined as the S2 CL at which both S2 stimuli did not lead to a subsequent ventricular action potential. Repetitive high-rate burst pacing with CL of 60–30 ms was applied to test inducibility of ventricular arrhythmias. Each CL was repeated twice to ensure reproducibility.

#### ECG and MAP analysis

ECG recordings were used to determine heart rate. 10 consecutive P-P intervals were measured and averaged for calculation of heart rate.

Signal quality criteria of MAP recordings encompassed a stable baseline and typical murine MAP morphology with a fast upstroke and rapid continuous repolarization without a plateau phase [24]. Action potential duration was analyzed at 90% repolarization. Peak of the action potential (AP) was defined as complete depolarization or 0% repolarization [25, 26]. 100% repolarization of the AP was measured during electrical diastole [25, 26]. APD_90_ was defined as the time difference between the peak of the AP and the time point of 90% repolarization.

#### Arrhythmia analysis

All ECG and MAP recordings were reviewed for arrhythmias with focus on monomorphic and polymorphic ventricular tachycardia and ventricular fibrillation. Ventricular tachycardia was defined as more than 5 consecutive ventricular beats with a shorter CL as sinus rhythm CL.

#### Data acquisition

All signals were recorded by a multichannel recorder and stored on a removable heart disc (Hugo-Sachs, March-Hugstetten, Germany). ECG and MAP recordings were analyzed by use of the “IsoHeart” software (Hugo-Sachs, March-Hugstetten, Germany) and a custom-made analysis software for determination of MAP duration.

### Statistical analysis

The SPSS software for Windows, release 18.0.0 (SPSS, Chicago, Illinois) was applied for statistical analysis. The Mann-Whitney-U-test and the one-way ANOVA- test were used to assess statistical differences regarding APD_90_, membrane potential, ventricular ERP, heart rate and inducibility of ventricular arrhythmias. Differences are considered significant at p < 0.05. Values are depicted as mean ± SEM.

## Results

### Cellular electrophysiology

#### APD_90_ is unchanged in cardiomyocytes from CVB3+ as compared to WT mice

**Fig 1:**
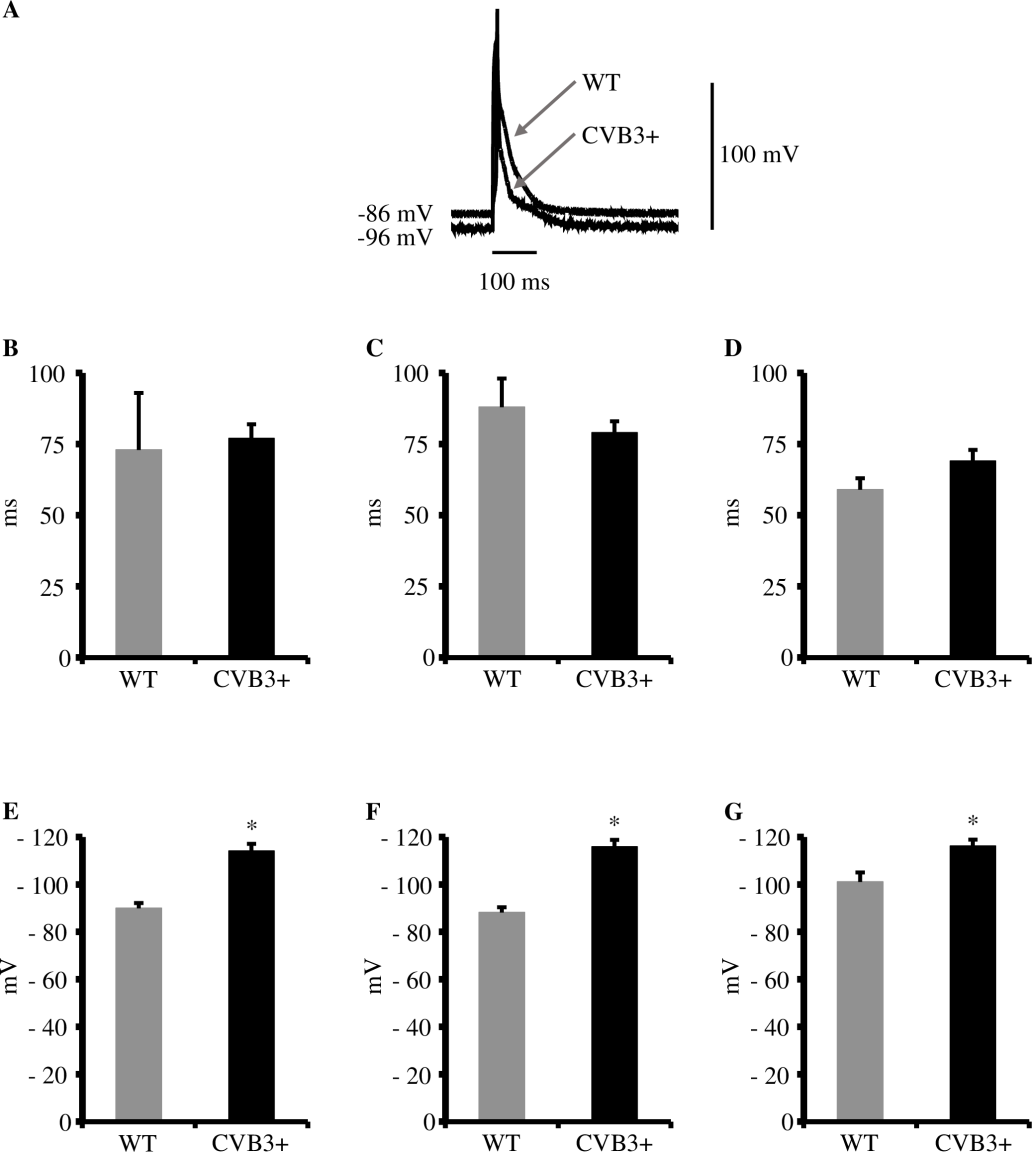
Action potential duration (APD_90_) and resting membrane potential in WT and CVB3+ cardiomyocytes. (A) Recordings of cellular action potentials from WT and CVB3+ cardiomyocytes. Action potential duration was unaltered between both genotypes. mV = millivolt, ms = milliseconds. (B) APD_90_ in WT and CVB3+ cardiomyocytes at 1 Hz stimulation. WT n = 7; CVB3+ n = 14. n = number of cardiomyocytes. (C) APD_90_ in WT and CVB3+ cardiomyocytes at 2 Hz stimulation. WT n = 7; CVB3+ n = 14. (D) APD_90_ in WT and CVB3+ cardiomyocytes at 5 Hz stimulation. WT n = 7; CVB3+ n = 14. (E) Resting membrane potential in WT and CVB3+ cardiomyocytes at 1 Hz. WT n = 7; CVB3+ n = 14; *p<0.005. (F) Resting membrane potential in WT and CVB3+ cardiomyocytes at 2 Hz. WT n = 7; CVB3+ n = 14; *p<0.005. (G) Resting membrane potential in WT and CVB3+ cardiomyocytes at 5 Hz. WT n = 6; CVB3+ n = 14; *p<0.005.

In order to detect potentially proarrhythmic modulations of cellular electrophysiology we recorded action potentials from isolated cardiomyocytes. Interestingly, we found APD_90_ to be unchanged in CVB3+ myocytes as compared to WT at stimulation frequencies of 1,2 and 5Hz [APD_90_ at 1Hz, WT 73±20 ms, n = 7; CVB3+ 77±5 ms, n = 14; p = ns.] [APD_90_ at 2Hz, WT 88±10 ms, n = 7; CVB3+ 79±4 ms, n = 14; p = ns.] [APD_90_ at 5Hz, WT 59±4 ms, n = 7; CVB3+, 69±4 ms n = 14; p = ns.] (Fig 1A–1D).

#### Resting membrane potential is hyperpolarized in CVB3+ cardiomyocytes

However, we found the resting membrane potential to be significantly hyperpolarized in CVB3+ cardiomyocytes at 1, 2 and 5 Hz. [1Hz: WT -90.0±2.2 mV, n = 7; CVB3+ -114.1±3.0 mV, n = 14; p<0.005] [2Hz: WT –88.2±2.2 mV, n = 7; CVB3+ –115.9±2.9 mV, n = 14; p<0.005] [5Hz: WT –101.1±4.0 mV, n = 6; CVB3+ –116.2±2.7 mV, n = 14; p<0.005] (Fig 1A and 1E–1G). In summary, we detected no proarrhythmic modulation in the kinetics of cellular action potentials from CVB3+ cardiomyocytes in comparison to WT under the conditions used here.

#### EADs are equally frequent and share a similar amplitude in cardiomyocytes from CVB3+ and WT mice

**Fig 2:**
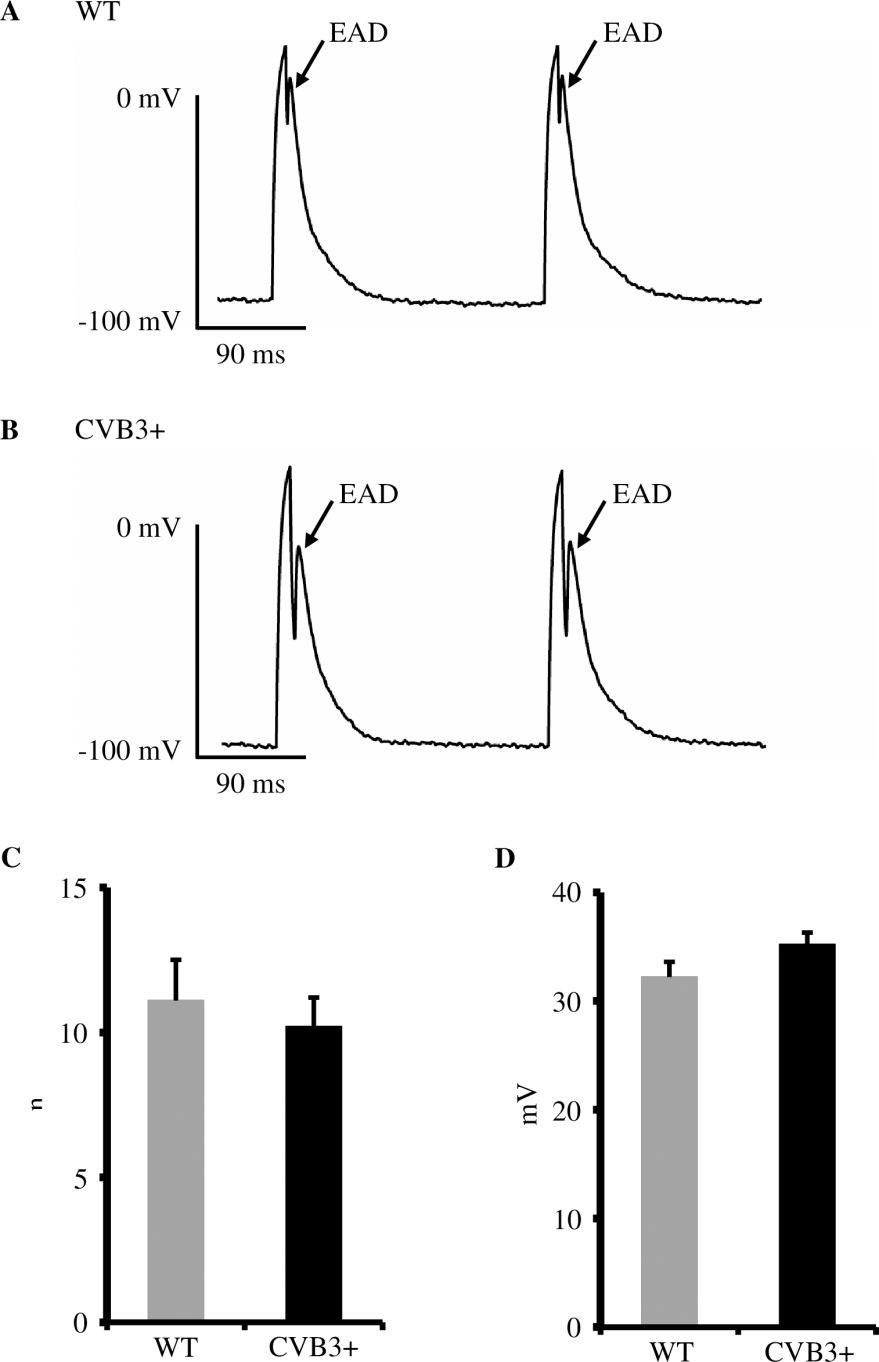
Early afterdepolarizations (EADs) in WT and CVB3 cardiomyocytes. (A) Representative trace of cellular action potentials and EADs in a WT cardiomyocyte. mV = millivolt, ms = milliseconds. (B) Representative trace of cellular action potentials and EADs in a CVB3+ cardiomyocyte. (C) Mean occurrence of EADs in WT and CVB3+ cardiomyocytes at 1,2 and 5 Hz. WT n = 4; CVB3+ n = 10. n = number of cardiomyocytes. (D) Mean amplitude of EADs in WT and CVB3+ cardiomyocytes. WT n = 4; CVB3+ n = 10.

In order to further investigate the potential arrhythmogenesis of CVB3 in ventricular myocardium, we analyzed action potentials from isolated cardiomyocytes for the occurrence and the amplitude of EADs and DADs. We primarily detected early and late EADs in both groups. We found no difference in the occurrence and amplitude of EADs in recordings from isolated cardiomyocytes from WT and CVB3+ mice at 1,2 and 5 Hz [WT 11.1±1.4 afterdepolarizations, n = 4; CVB3+ 10.2±1 afterdepolarizations, n = 10; p = ns.] [WT 32.2±1.4 mV, n = 4; CVB3+ 35.2±1.1 mV, n = 10; p = ns.] (Fig 2A–2D).

### Whole heart Langendorff-perfusion

#### Action potential duration

**Fig 3:**
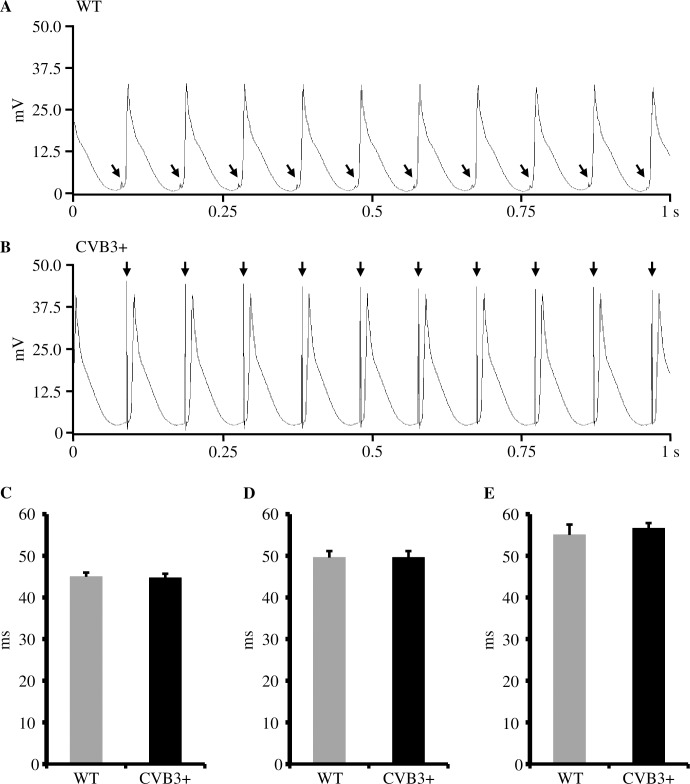
Monophasic action potentials and action potential duration (APD_90_) in WT and CVB3+ whole hearts. (A) Representative recording of monophasic action potentials in a Langendorff-perfused WT whole heart at pacing with S1 = 100 ms (ms = milliseconds). mV = millivolt, s = seconds. ↓ = electrical stimulus artefact. (B) Representative recording of monophasic action potentials in a Langendorff-perfused CVB3+ whole heart at pacing with S1 = 100 ms. (C) APD_90_ in Langendorff-perfused WT and CVB3+ hearts at pacing with S1 = 100 ms. WT n = 7; CVB3+ n = 7. n = number of Langendorff-perfused hearts. (D) APD_90_ in Langendorff-perfused WT and CVB3+ hearts at pacing with S1 = 120 ms. WT n = 7; CVB3+ n = 7. (E) APD_90_ in Langendorff-perfused WT and CVB3+ hearts at pacing with S1 = 150 ms. WT n = 6; CVB3+ n = 7. Within each genotype, we found a heart rate dependent shortening of ADP_90_ with increasing heart rate. (WT ADP_90_ at: S1 = 100 ms vs. S1 = 150 ms, p = 0.014; CVB3+ ADP_90_ at: S1 = 100 ms vs. S1 = 150 ms, p = 0.001).

We determined action potential duration in order to test, whether chronic CVB3 expression may cause alteration of action potential duration and thus may generate a proarrhythmic substrate [2]. Fixed-frequent S1 ventricular pacing showed similar action potential durations at 90% repolarization (ADP_90_) between WT and CVB3+ whole hearts. [ADP_90_ S1 = 100 ms; WT 45.0±1.5 ms, n = 7; CVB3+ 44.7±1.8 ms, n = 7; p = 0.71] [ADP_90_ S1 = 120 ms; WT 49.6±1.6 ms, n = 7; CVB3+ 49.6±1.5 ms, n = 7; p = 1] [ADP_90_ S1 = 150 ms; WT 55.0±2.1 ms, n = 6; CVB3+ 56.6±1.3 ms, n = 7; p = 0.84]. Within each genotype, we found a heart rate dependent shortening of the ADP_90_ with increasing heart rate. [WT ADP_90_ at: S1 = 100 ms vs. S1 = 150 ms, p = 0.014; CVB3+ ADP_90_ at: S1 = 100 ms vs. S1 = 150 ms, p = 0.001] (Fig 3A–3E).

#### Ventricular effective refractory period

**Fig 4:**
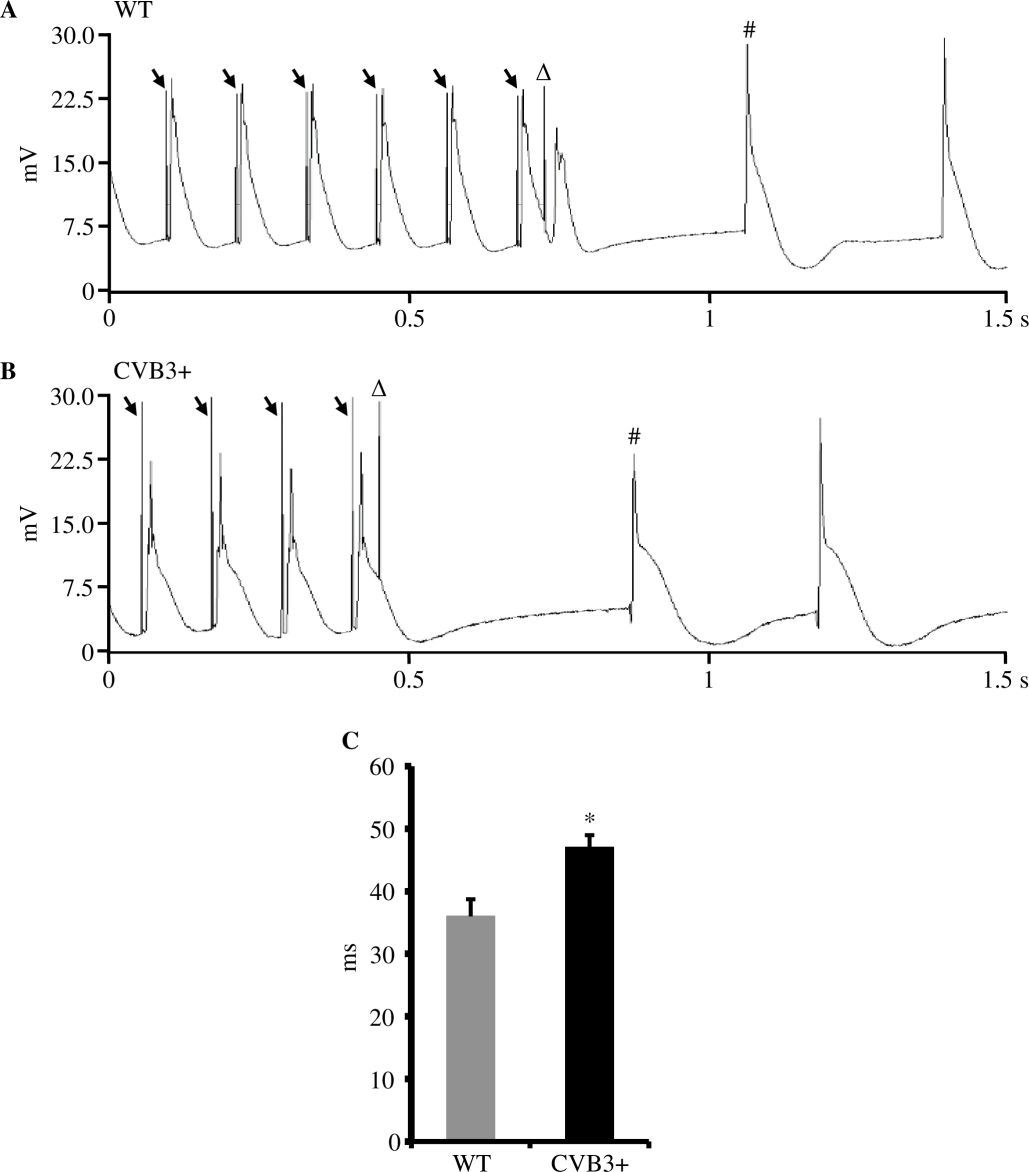
Ventricular effective refractory period (VERP) in WT and CVB3+ whole hearts. (A) Recording of S1S2 electrical stimulation protocol in a Langendorff-perfused WT whole heart. ↓ = S1 electrical stimulus artefact. S1 cycle length was 120 ms (ms = milliseconds). Δ = S2 extra-stimulus for determination of VERP. In this example, S2 had a coupling interval of 46 ms that caused a subsequent ventricular depolarization. # = first intrinsic ventricular action potential. mV = millivolt, s = second. (B) Recording of S1S2 electrical stimulation protocol in a Langendorff-perfused CVB3+ whole heart. S2 extra-stimulus (Δ) coupling interval was also 46 ms that did not cause a subsequent ventricular depolarization, indicating ventricular refractoriness (VERP). (C) VERP in Langendorff-perfused WT and CVB3+ whole hearts. WT n = 5; CVB3+ n = 7; *p = 0.018. n = number of Langendorff-perfused hearts.

In order to determine whether CVB3 expression may alter cardiac repolarization, we recorded the ventricular effective refractory period (VERP). In CVB3+ whole hearts, VERP was slight but significantly prolonged compared to WT hearts. [VERP, WT 36.0±2.7 ms, n = 5; CVB3+ 47.0±2.0 ms, n = 7; p = 0.018] (Fig 4A–4C).

#### Heart rate

**Fig 5:**
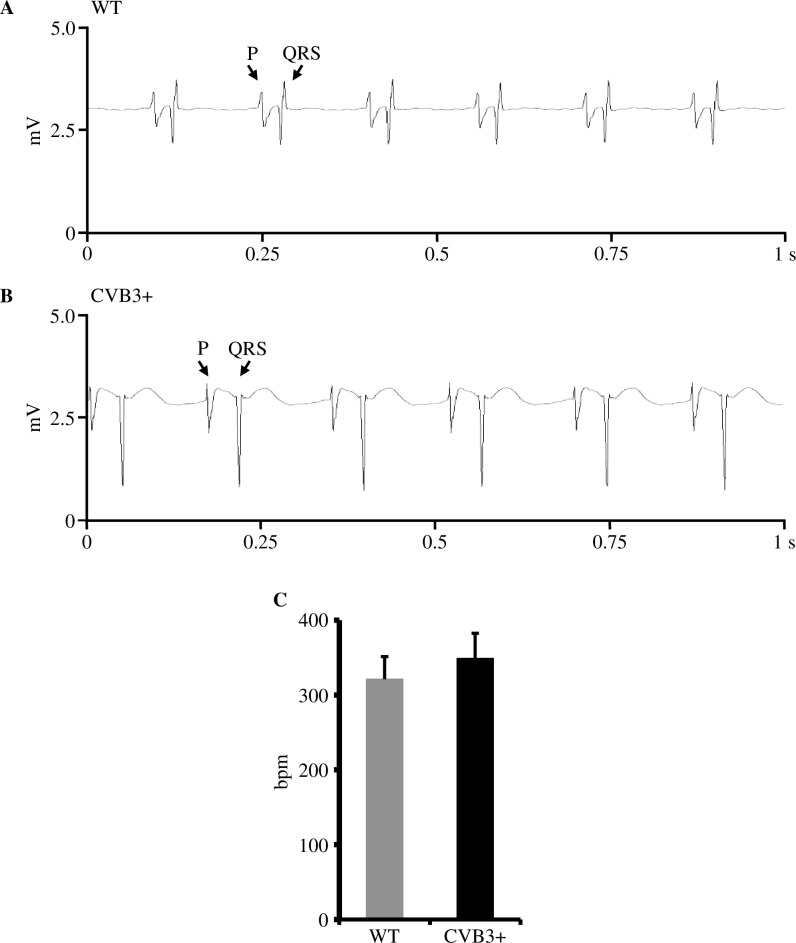
Resting heart rate in WT and CVB3+ whole hearts. (A) Representative recording of resting heart rate in a Langendorff-perfused WT whole heart. mV = millivolt, s = seconds, P = P wave, QRS = QRS complex. Heart rate is 382 bpm (bpm = beats per minute). (B) Representative recording of resting heart rate in a Langendorff-perfused CVB3+ whole heart. Heart rate is 347 bpm. (C) Mean resting heart rate in Langendorff-perfused WT and CVB3+ whole hearts. WT n = 7; CVB3+ n = 7. n = number of Langendorff-perfused hearts.

Further, we examined whether CVB3 expression may have an impact on pacemaker activity and resting heart rate. In Langendorff-perfused whole hearts, resting heart rate was not significantly different between both genotypes. [Heart rate (HR) in beats per minute (bpm); WT 321.1±30.2 bpm, n = 7; CVB3+ 348.9±33.5 bpm, n = 7; p = 0.81]. CVB3+ hearts showed only a trend to a minimal higher resting heart rate as WT (Fig 5A–5C).

#### Arrhythmia induction

Finally, we used electrical pacing protocols to study if CVB3 expression alters cardiac electrophysiology in a proarrhythmic manner leading to increased vulnerability to ventricular arrhythmias in CVB3+ murine hearts. Applied pacing protocols found no enhanced inducibility of ventricular arrhythmias in CVB3+ (n = 5) Langendorff-perfused whole hearts compared to WT (n = 5).

## Discussion

The present study was conducted to study the electrophysiological properties in transgenic mice expressing a full length CVB3 cDNA mutant (CVB3ΔVP0). This mouse model mimics persistence of the CVB3 genome with low levels of CVB3 genome replication and protein expression resembling chronic viral myocarditis or enterovirus-induced dilated cardiomyopathy. In the present study we characterized cellular and whole heart electrophysiology in this CVB3+ mouse model and showed that several cardiac electrophysiological parameters were not altered by CVB3 expression compared to WT. Therefore, generation of arrhythmias in chronic CVB3 myocarditis seems not to depend on altered cardiac electrophysiology but rather on other mechanisms such as chronic inflammation and myocardial fibrosis.

A previous study by Wessely et al. also investigated restricted viral replication with low level expression of CVB3 proteins in a mouse model [18]. In these transgenic mice, replication of the CVB3 genome was under the control of the cardiac myocyte-specific myosin light chain-2v (MLC-2v) promoter [18]. Wessely et al. demonstrated in their study, that their mice developed features of dilated cardiomyopathy as myocardial interstitial fibrosis, impaired excitation–contraction coupling, left ventricular dilatation and reduced systolic left ventricular function [18]. In addition, these mice showed reduced amplitude of [Ca^2+^]_i_ transient and unaltered I_Ca_ current density [18].

### Cellular electrophysiology

#### Action potential duration

We found no difference in APD_90_ using stimulation frequencies of 1,2 and 5 Hz in CVB3+ cardiomyocytes compared to WT cardiomyocytes (Fig 1A–1D). Therefore, persistent CVB3 infection and low level genome expression seem not to have a significant effect on depolarizing and repolarizing ion currents, which is in contrast to acutely CVB3 infected cultured cells in which an alteration of I_Ks_, I_Kr_ and I_CaL_ was found [2]. As we detected no prolonged APD_90_ in CVB3+ cardiomyocytes, it may be assumed that I_Ca_ current was not significantly affected, which is in accordance with the finding of Wessely et al. in a previous CVB3+ myocarditis mouse model which demonstrated unaltered I_Ca_ current density in their CVB3+ cardiomyocytes [18].

#### Resting membrane potential

Furthermore, we investigated a potential influence of altered cardiac ion channel properties on membrane potential in isolated cardiomyocytes from CVB3+ mice. Interestingly, we found a solid reduction of the resting membrane potential in cardiomyocytes from CVB3+ mice compared to wildtype mice (Fig 1A and 1E–1G), which may even represent a potential protective electrophysiological feature against ventricular arrhythmias.

#### Afterdepolarizations

In accordance to our APD_90_ data we were not able to detect a difference in the mean occurrence of early and late EADs in ventricular cardiomyocytes between both genotypes (Fig 2A–2C). Early EADs are associated with the reactivation of Ca^2+^ channels [27]. Since the L-type Ca^2+^ current seems not to differ in CVB3+ cardiomyocytes from wildtype cardiomyocytes there is no obvious basis for proarrhythmic I_Ca_ alterations that could trigger spontaneous activity in CVB3+ cells. We analyzed the amplitude of all EADs in both groups and found no difference between CVB3+ and WT cardiomyocytes (Fig 2D), making occurrence of ventricular arrhythmias in CVB3+ hearts less likely.

In summary, based on our cellular electrophysiologic results, we conclude that the combination of a lower resting membrane potential with any EADs being equally frequent and unchanged in amplitude in CVB3+ cardiomyocytes, the threshold for L-type I_Ca_ or I_Na_ is less likely surpassed in CVB3+ mice as compared to wildtype mice, making spontaneous activity and ventricular arrhythmias altogether less likely. A similar mechanism has been shown by Pott et al. where altered amplitudes of DADs and EADs were associated with an antiarrhythmic effect in NCX-KO mice [28]. However, it remains unclear which underlying mechanism mediates this difference in membrane potential in CVB3+ cardiomyocytes or whether the virus proteins themselves cause this alteration in CVB3+ cardiomyocytes.

### Beating Langendorff-perfused whole hearts

In order to validate the impact of our cellular electrophysiological results we studied whole CVB3+ hearts by using a whole heart Langendorff-setup.

#### Action potential duration

Compared to our cellular electrophysiological experiments, we also determined ventricular action potential durations at 90% repolarization (ADP_90_) in CVB3+ whole hearts with our Langendorff-setup. We could demonstrated that APD_90_, in accordance with our cellular data from cardiomyocytes, was not significantly different between CVB3+ and WT whole-hearts and that both genotypes showed preserved heart rate dependent shortening of ADP_90_ with increasing heart rates (Fig 3A–3E).

As already mentioned in the previous section, persistent CVB3 low level expression seems not to have an impact on depolarizing and repolarizing ion channels, which is supported by a previous CVB3 mouse model which also presented unaltered I_Ca_ current density in CVB3 cardiomyocytes [18]. Further, impact of chronic CVB3 expression on cardiac ion channels, which is reflected indirectly by ADP_90_, seems to be negligible and is in contrast to acute CVB3 infection which caused alterations of I_Ks_, I_Kr_ and I_CaL_ in cultured cells [2].

#### Ventricular effective refractory period

Further, we studied whether CVB3+ expression may have an effect on ventricular repolarization. Our study showed that ventricular effective refractory period (VERP) was slightly but significantly prolonged in CVB3+ hearts compared to WT hearts (Fig 4A–4C). This finding may imply an altered reactivation kinetic of depolarizing currents. In addition, a prolonged VERP may even contribute to suppression of arrhythmias.

#### Heart rate

As known from the clinical setting, patients with advanced stages of heart failure have an increased resting heart rate, which may be due to altered cardiac ion channel properties [29]. Therefore, we investigated whether CVB3+ expression may alter function of the cardiac pacemaking system. We found only a slightly increased resting heart rate in CVB3+ hearts compared to WT hearts without reaching statistical significance (Fig 5A–5C) which may suggest that CVB3+ seem not to affect function of the cardiac pacemaking system.

Upstroke of the action potential of sinus node pacemaker cells is mainly generated by the funny channel I_f_ and I_CaL_ [30]. As we found no difference in resting heart rate, it may be suggested that I_f_ and I_CaL_ were not impaired by CVB3 expression.

#### Arrhythmia induction

Patients with acute and chronic CVB3 myocarditis or cardiomyopathy may suffer from ventricular arrhythmias or sudden cardiac death [12]. Therefore, we investigated whether CVB3+ hearts were vulnerable to ventricular arrhythmias. In Langendorff-perfused hearts, we applied electrical pacing protocols for studying induction of ventricular arrhythmias. No ventricular arrhythmia could be induced in CVB3+ and WT hearts. On the one hand, this finding is of particular interest and unexpected, as we would expect an increased tendency to ventricular arrhythmias in CVB3+ hearts based on our clinical experience [31]. On the other hand, our study found mainly unaltered electrophysiological properties and only little changed parameters which might even protect against ventricular arrhythmias in CVB3+ hearts, as prolonged ventricular effective refractory period and a more negative resting membrane potential in CVB3+ mice, may act as an antiarrhythmic feature [32, 33], making the heart less vulnerable to ventricular arrhythmias. Based on our findings, it may be suggested that persistent low level CVB3 expression seem not to have a proarrhythmic impact on cardiac electrophysiological properties and generation of arrhythmias. Therefore, it may be speculated that other mechanism like myocardial inflammation and myocardial fibrosis may mainly contribute to arrhythmogenesis in patients with CVB3 infection, as we know that virus myocarditis is associated with myocardial inflammation and myocardial injury resulting in myocardial fibrosis which may support arrhythmias [29, 34]. A previous murine study showed that low level CVB3 gene expression in myocytes in a pattern similar to that in hearts of patients with persistent viral infection may cause myocytopathic effects [17]. Further studies confirmed the finding that CVB3 replication may induce myocardial injury due to apoptosis and necrosis of cardiomyocytes [1, 35]. In summary, these studies may support the hypothesis that low level CVB3 gene expression induces apoptosis and necrosis of myocytes and inflammation, which subsequently leads to myocardial fibrosis and scars. These regions of myocardial fibrosis and scars may represent a proarrhythmic substrate and a potential mechanism of arrhythmia induction during low level CVB3 gene expression in chronic CVB3 myocarditis. In addition, this potential arrhythmic mechanism in CVB3 viral cardiomyopathy would be similar to that of patients with coronary heart disease and myocardial infarction, as occlusion of coronary vessels causes myocardial necrosis with subsequent formation of myocardial scars which are regions of a proarrhythmic substrate for formation of macro-reentrant waves of ventricular arrhythmias [36].

### Sex based differences in cardiac electrophysiology

Murine studies demonstrated that a stimulatory impact of androgens on the ultra-rapid delayed rectifier K^+^ current (I_Kur_) with the underlying Kv1.5 channel is responsible for gender differences in cardiac repolarization [37–39]. Androgens cause an increased I_Kur_ current and enhanced Kv1.5 expression with subsequent shortening of ventricular repolarization in male mice compared to female mice [37–39]. Further, a study using male mice of different strains revealed that testosterone levels are variable between common mice strains (CD-1, C57BL/6, C3H and FVB) and that male C57BL/6 mice have very low testosterone levels compared to normal testosterone levels in the other mouse strains [37]. The whole-cell voltage clamp technique in isolated cardiomyocytes of C57BL/6 mice demonstrated that the current density of I_Kur_ in male C57BL/6 mice was similar to that of female C57BL/6 mice due to the very low testosterone level of the male C57BL/6 mice [37]. In our study, we used whole hearts and cardiomyocytes form male C57BL/6 mice only. Therefore, based on the study of Brouillette et. al. [37] in male and female C57BL/6 mice, we would not expect sex based differences of our electrophysiological results derived from CVB3+ and WT C57BL/6 mice.

### Translational perspective

A wide range of patients with CVB3 viral myocarditis are clinically unapparent or present mild symptoms with complete convalescence, whereas a certain proportion of patients suffer from rapid progress and severe cardiac dysfunction due to acute or chronic viral myocarditis leading to cardiomyopathy, ventricular arrhythmias or sudden cardiac death. Our study may imply that in patients with chronic CVB3 myocarditis, electrophysiological properties seem mainly not to be affected by the CVB3 genome in a proarrhythmic manner and thus contribute rather not to arrhythmogenesis. As low level CVB3 gene expression in myocytes can cause cytopathic effects [17] with subsequent myocardial inflammation and fibrosis which may cause a proarrhythmic substrate for re-entrant arrhythmias [24], it may be suggested that these histopathological alterations are predominantly responsible for generation of arrhythmias in patients with chronic CVB3-induced cardiomyopathy. This in turn may represent a potential therapeutic target in CVB3-infected patients. Administration of anti-inflammatory and immunosuppressive drugs or angiotensin converting enzyme inhibitors may suppress myocardial inflammation and fibrosis and thus decrease vulnerability to ventricular arrhythmias and sudden cardiac death [1, 29].

Further, our study might contribute to the understanding of differences of acute and chronic CVB3 myocarditis, as a study in *Xenopus laevis* oocytes with acute CVB3 infection found acute alterations of cardiac ion channels I_Ks_, I_Kr_ and I_CaL_ which might encourage arrhythmias [2]. These findings seem to be in contrast to chronic CVB3 myocarditis, as we could not detect significant proarrhythmic alterations of cardiac electrophysiology.

## Limitations

As our study used a murine model, obtained results cannot uncritically be transferred into the clinical context due to partial different electrophysiological properties between mice and man [24, 40–42]. However, several studies showed that mouse models can mimic human cardiac arrhythmias, myocardial infarction and heart failure, thus contributing to our knowledge of cardiac diseases [24, 43–46].

Currently, we can only confirm CVB3+ expression in our homozygous CVB3+ transgenic mice by using nested-PCR without having quantitative data of the CVB3+ expression level, which is subject of a current study. Furthermore, we cannot present imaging data concerning myocardial function of CVB3+ mice, which is also subject of a current study.

Our study suggests that low level CVB3 expression does not promote VA by altered cardiac electrophysiology in chronic myocarditis. As previous studies showed that low level CVB3 gene expression in myocytes may lead to cytopathic effects [17, 18] with subsequent chronic myocardial inflammation and fibrosis [18] which may cause a proarrhythmic substrate for re-entrant VA [24], it may be suggested that these mechanisms may account for arrhythmias observed in patients with chronic enteroviral myocarditis. As we used mice at an age of 24 weeks with low level CVB3 expression, it may be speculated that myocardial inflammation, fibrosis and reduced left ventricular ejection fraction were only slightly present at this age of mice, which may explain that we could not detect VA. It would be interesting to repeat electrophysiological measurements at a higher age of CVB3+ mice when chronic myocardial inflammation may have resulted in a greater extend of myocardial fibrosis, scars and impaired left ventricular ejection fraction. If electrophysiological parameters would then also have antiarrhythmic properties or would be not altered compared to WT mice, this may be considered as an additional confirmation of the hypothesis that chronic myocardial inflammation, scars and reduced left ventricular ejection fraction may be the predominant mechanisms for VA in chronic CVB3 virus-induced cardiomyopathy.

## Conclusions

Our study in transgenic mice showed that chronic low level CVB3 expression seems not to have a significant impact on cardiac ion channels and cardiac electrophysiology. Our findings may support the hypothesis that other mechanisms like chronic myocardial inflammation over a longer period of time with necrosis and subsequent myocardial scars may predominantly contribute to VAs in chronic CVB3 myocarditis. At the age of mice used in the current study, extent of myocardial inflammation with necrosis and myocardial scars resulting in reduced left ventricular function seems to be less pronounced which may explain that we could not detect VA.

In summary, generation of arrhythmias in patients with chronic CVB3-induced cardiomyopathy probably does not rely on altered cardiac electrophysiology but rather on chronic myocardial inflammation over a longer period of time with subsequent necrosis and development of myocardial scars as a proarrhythmic substrate for reentrant arrhythmias. Based on this hypothesis, use of anti-inflammatory and immunosuppressive drugs or angiotensin converting enzyme inhibitors may be promising therapeutic strategies to suppress myocardial remodeling and generation of pro-arrhythmic myocardial scars.

## Supporting information

S1 Table Dataset. The S1 Table contains all original data of the study.
(XLSX)
